# Hydrogen–methane breath testing results influenced by oral hygiene

**DOI:** 10.1038/s41598-020-79554-x

**Published:** 2021-01-08

**Authors:** Sharon Erdrich, Edwin C. K. Tan, Jason A. Hawrelak, Stephen P. Myers, Joanna E. Harnett

**Affiliations:** 1 Faculty of Medicine and Health, School of Pharmacy, The University of Sydney, Sydney, NSW 2006 Australia; 2 School of Pharmacy and Pharmacology, College of Health and Medicine, University of Tasmania, Private Bag 26, Hobart, TAS 7001 Australia; 3 NatMed-Research Unit, Southern Cross University, PO Box 157, Lismore, NSW 2480 Australia

**Keywords:** Microbiology, Gastroenterology, Health care

## Abstract

The measurement of hydrogen–methane breath gases is widely used in gastroenterology to evaluate malabsorption syndromes and bacterial overgrowth. Laboratories offering breath testing provide variable guidance regarding oral hygiene practices prior to testing. Given that oral dysbiosis has the potential to cause changes in breath gases, it raises concerns that oral hygiene is not a standard inclusion in current breath testing guidelines. The aim of this study was to determine how a pre-test mouthwash may impact hydrogen–methane breath test results. Participants presenting for breath testing who had elevated baseline gases were given a chlorhexidine mouthwash. If a substantial reduction in expired hydrogen or methane occurred after the mouthwash, breath samples were collected before and after a mouthwash at all breath sample collection points for the duration of testing. Data were evaluated to determine how the mouthwash might influence test results and diagnostic status. In 388 consecutive hydrogen–methane breath tests, modifiable elevations occurred in 24.7%. Administration of a chlorhexidine mouthwash resulted in significantly (*p* ≤ 0.05) reduced breath hydrogen in 67% and/or methane gas in 93% of those consenting to inclusion. In some cases, this modified the diagnosis. Mean total gas concentrations pre- and post-mouthwash were 221.0 ppm and 152.1 ppm (*p* < 0.0001) for hydrogen, and 368.9 ppm and 249.8 ppm (*p* < 0.0001) for methane. Data suggest that a single mouthwash at baseline has a high probability of returning a false positive diagnosis. Variations in gas production due to oral hygiene practices has significant impacts on test interpretation and the subsequent diagnosis. The role of oral dysbiosis in causing gastrointestinal symptoms also demands exploration as it may be an underlying factor in the presenting condition that was the basis for the referral.

## Introduction

Hydrogen–methane breath testing is a widely used diagnostic tool, based on the science that these gases are by-products of saccharide fermentation by gut microorganisms, rather than human metabolism.

Glucose, lactose, and fructose are normally absorbed in the small intestine. Increased gas production following their ingestion is associated with malabsorption or premature fermentation due to excessive bacteria in the small intestine. Hydrogen and methane gas are absorbed from the gastrointestinal tract, exhaled via the lungs and are thus measurable in breath. Increased gas production following ingestion of fructose or lactose are used to detect malabsorption of carbohydrates. Similarly, increases in expired breath gases in response to ingestion of non-digestible lactulose, or glucose are used to predict small intestinal bacterial overgrowth (SIBO)^1–4^.

In-depth discussion on the methodology and interpretation of breath testing was published in 2009^5^ and a shorter summary in 2011^6^. The concept that expired breath gases may be influenced by the oral microbiome is not yet an integrated component of breath testing guidelines, even though oral dysbiosis has been implicated in oral and non-oral diseases^7^.

To improve the accuracy of breath testing, a 1-day preparatory low-residue diet and water-only overnight fast is required. After collection of a baseline breath sample, the challenge substrate (usually lactulose, glucose, fructose, or lactose) is administered, and subsequent samples of breath are collected at pre-determined intervals for the duration of the test period. Interpretation of results varies between testing facilities, which is an issue that the North American Consensus sought to address in their 2017 guideline^8^.

Neither this guideline^8^ nor any other in the reviewed breath testing literature, sets a clear definition for normal baseline gases. In practice, a variety of breath patterns is seen^9–13^, the meaning of which is unclear. These include markedly elevated hydrogen and/or methane, which may remain high throughout testing or may subsequently decrease, sometimes immediately following ingestion of the test substrate. Proposed contributions to this elevation include inadequate preparation for the test or delayed colonic transit time, resulting in fermentative residue in the intestines. Such basal elevations may confound the results of challenge testing, as the gas level may naturally decrease due to being either excreted in flatus or stool, or otherwise metabolized during the course of the breath test. Such changes may mask increases occurring due to fermentation of the test substrate. At least one researcher suggests an elevated baseline indicates poor preparation and that testing should be abandoned^6^. The role of oral or respiratory bacteria in elevated baselines has to date not been explored.

A recent survey of clinical practices found inconsistency amongst breath testing centres as to the definition of elevated levels of baseline hydrogen, methane or combined gases^14^. Possible reasons for this inconsistency include poor test–retest reliability for these gases and that oral hygiene may impact baseline measurements.

The 2009 Rome Consensus on hydrogen breath testing^5^ includes the statement “consider mouthwash with chlorhexidine solution before substrate ingestion”. This is open to interpretation in terms of whether to apply the recommendation, as well as when (relative to commencing testing) it should be undertaken. Ghoshal’s 2011 review^6^ also recommended antiseptic mouth wash, without detailing that this should be immediately prior to sample collection. Oral hygiene was not specified in either the 2017 North American Consensus^8^, nor in an updated guideline published in 2020^15^. Our small survey of current practices revealed a range of approaches to the preparation of the oral cavity before undertaking breath testing^14^.

Therefore, this study aimed to evaluate the influence of oral hygiene on baseline measures of hydrogen and methane and whether continuance of oral cleaning practice for the duration of the breath test alters the test outcome and subsequent diagnosis.

## Methods

*Subjects* Adult patients (≥ 18 years of age), presenting for hydrogen–methane breath testing for saccharide malabsorption or determination of likelihood of SIBO were screened for adherence to test preparation, including non-smoking, avoidance of exercise and compliance with a 1-day low residue diet excluding all foods except white rice, fish, chicken, eggs, white (dairy-free) bread, clear broths and plain black tea or coffee. Small amounts of cooking oils, salt and pepper were permitted. This was followed by a minimum 12-h water-only overnight fast.

*Protocol* Subjects were instructed to breathe normally (avoiding deep inspiration and hyperventilation) into a simple alveolar collection device with syringe port. A 30cc volume of end expiratory air was collected and immediately analyzed using a gas chromatograph dedicated to the detection of hydrogen, methane, and carbon dioxide (CO_2_) in air (Model SC, Quintron Instrument, Milwaukee, WI, USA). Data were reported in parts per million (ppm) and corrected for CO_2_ content. The accuracy of the detector was ± 3 ppm.

All subjects with ≥ 10 ppm of hydrogen or methane at baseline were administered a mouthwash using 10 mL of a chlorhexidine (1.2 mg/mL) mouthwash (Colgate Savacol) with instructions to move the mouthwash around the mouth for 20–30 s, forcing it between teeth and gargling before spitting it out and rinsing the mouth with water. A subsequent breath sample was then collected and analyzed for comparison to the pre-mouthwash values. Where a reduction of either ≥ 10 ppm or ≥ 25% of the individual or combined gases following administration of the mouthwash was recorded, subjects were assigned to the following procedure:

- Administration of the test substrate as prescribed (i.e., lactulose 10 g, glucose 75 g, fructose 25 g or lactose 25 g) following baseline sampling.
- Subsequent breath samples were collected for retesting every 20 min for lactulose, every 15 min for glucose and every 30 min for fructose and lactose.
- At each retest time-point, breath sample collection was followed by a repeat mouthwash as per the above procedure, followed by immediate collection of another breath sample.
- Breath samples were collected at baseline (fasting) up to a maximum of 180 min and analyzed within 3 min of collection (Table 1).

**Table 1:** Conducting breath tests using various substrates^8^.

Substrate	Dose	Intervals breath samples collected	Duration of testing	Explanation
Glucose	75 g	15 min	2 h^a^	Glucose is rapidly absorbed in proximal small intestine
Lactulose	10 g	20 min	2 h	2 h (3 h to capture expected colonic increase^16^)
Lactose	25 g	30 min	3 h^a^	Any increase indicates malabsorption
Fructose	25 g	30 min	3 h^a^	Any increase indicates malabsorption

^a^Test may be terminated earlier, if a peak above baseline is demonstrated.

### Data analysis

All data was entered into Microsoft Excel, and IBM Statistical Package for the Social Sciences (SPSS) version 26 and GraphPad Prism version 7 were used for analysis.

Paired samples t-tests were used to compare mean area-under-the-curve (AUC) between pre and post mouthwash gases. Cochran’s Q test and post-hoc McNemar’s tests with Bonferroni correction were used to compare between the three methods as outlined in Table 2:

1. No (pre) mouthwash data (A and C);
2. Single mouthwash at baseline only (B and C); and
3. Always (post) mouthwash (B and D).

**Table 2:**
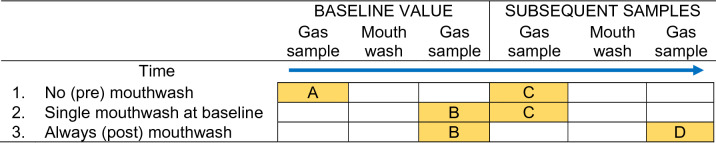
Graphical presentation of study protocol and data analysis.

Analysis of each method was conducted in consideration of each of the following diagnostic criteria within 90 min of ingestion of the test substrate (i.e. at 80 min for lactulose-challenge testing):

A. Increase in hydrogen ≥ 20 ppm
B. Increase in methane ≥ 12 ppm
C. Increase in combined hydrogen + methane ≥ 15 ppm
D. Increase of either hydrogen or methane ≥ 10 ppm between any two consecutive samples.

We also evaluated the data for variations in the time a subject first meets any, or each of these diagnostic criteria depending on the mouthwash protocol applied.

Further analyses were performed to assess change in diagnosis status following three scenarios (refer to Table 2):

1. Status based on evaluation of pre-mouthwash data (A–C), compared to post-mouthwash data (B–D).
2. Status based on evaluation of pre-mouthwash data (A–C), compared to status based on mouthwash at baseline only (B–C).
3. Status based on evaluation of post-mouthwash data (B–D), compared to status based on mouthwash at baseline only (B–C).

For each of these, “mouthwash at baseline only” increases are calculated based on the breath sample collected after a mouthwash (B). Analyses, using Cochran’s Q test were run for breath samples (C) at time points 1 and 4, where sample 0 is the baseline.

### Ethical approval

All procedures involving human participants were in accordance with the ethical standards of the institutional research committee and with the 1964 Helsinki declaration and its later amendments or comparable ethical standards. Ethical approval was obtained from the University of Sydney Human Research Ethics Committee (Ref. No. 2019/583).

### Informed consent

Informed consent was obtained from all individual participants included in the study.

## Results

Eligibility and consent for inclusion from 64 subjects provided data from 69 breath tests. Five were repeat tests on subjects who had previously been tested and are included twice in Table 3, as at least one had a birthday between tests.

**Table 3:** Baseline characteristics of cohort (n = 69).

Gender	n = (%)	Age		Weight (kg)	Baseline gases ppm mean (SD)	
		Range	Mean (SD)	Mean (SD)	Hydrogen	Methane
Female	49 (71.0)	18–72	42.3 (14.5)	63.1 (11.8)	9.6 (9.0)	27.8 (22.7)
Male	20 (29.0)	18–67	43.8 (16.3)	83.6 (15.6)	10.85 (16.1)	26.7 (21.0)

Baseline gases of ≥ 10 ppm of either hydrogen or methane were observed in 96 of 388 consecutive tests (24.7%). Consent for inclusion in our study provided 69 test results for analysis.

In forty-two baseline measurements (60.8% of the included 69), fasting methane gas was ≥ 20 ppm, and 56 (81.1% of the included 69) met the inclusion threshold of ≥ 10 ppm at baseline. In two subjects (2.9%) no differences in expired gases were observed after the initial sample, and in two (2.9%) the initial differences were lost by the third sample after baseline.

Lactulose was the test substrate in 63 tests (91.3%), glucose in four (5.8%), with fructose and lactose being used once each (1.4%). When comparing pre-mouthwash gas values to those obtained after the mouthwash, significant reductions in at least one gas was seen in 65 of 69 tests. Upon evaluating which gas was altered due to the mouthwash, hydrogen was reduced in 46/69, methane in 64/69. Both gases were markedly reduced in 45/69 tests, with notably greater reductions in methane seen. Table 4 presents the number of tests meeting three distinct *p* values for differences in concentrations of expired breath gases obtained as the result of mouth washing over the course of the testing procedure, with 63.7% of methane reductions meeting < 0.001 significance.

**Table 4:** Number of tests (%) with gas differences meeting various statistical significance cut-offs following mouthwash with chlorhexidine.

Gas	Total (%)*	*p* ≤ 0.05	*p* ≤ 0.01	*p* ≤ 0.001
Hydrogen	46 (66.7)	14 (20.3)	25 (36.2)	7 (10.1)
Methane	64 (92.7)	5 (7.0)	15 (21.7)	44 (63.7)
Hydrogen + methane	45 (65.2)	45 (65.2)	0	0

*At least *p* ≤ 0.05.

A marked difference was observed in mean AUC for hydrogen and methane gas over the duration of breath testing (Table 5).

**Table 5:** Comparison of area-under the curve (AUC) pre versus post-mouthwash (n = 69).

	Mean AUC (SD)		Mean difference (SD)	*p* value^a^
	Pre-mouthwash	Post-mouthwash		
Hydrogen	65.5 (61.9)	45.3 (39.2)	20.2 (28.1)	< 0.001
Methane	112.4 (91.2)	76.2 (61.7)	36.2 (35.1)	< 0.001
Hydrogen + methane	177.9 (90.3)	121.9 (62.5)	56.0 (40.8)	< 0.001

^a^Paired t-test.

*AUC* area under the curve; *SD *standard deviation.

**Figure 1:**
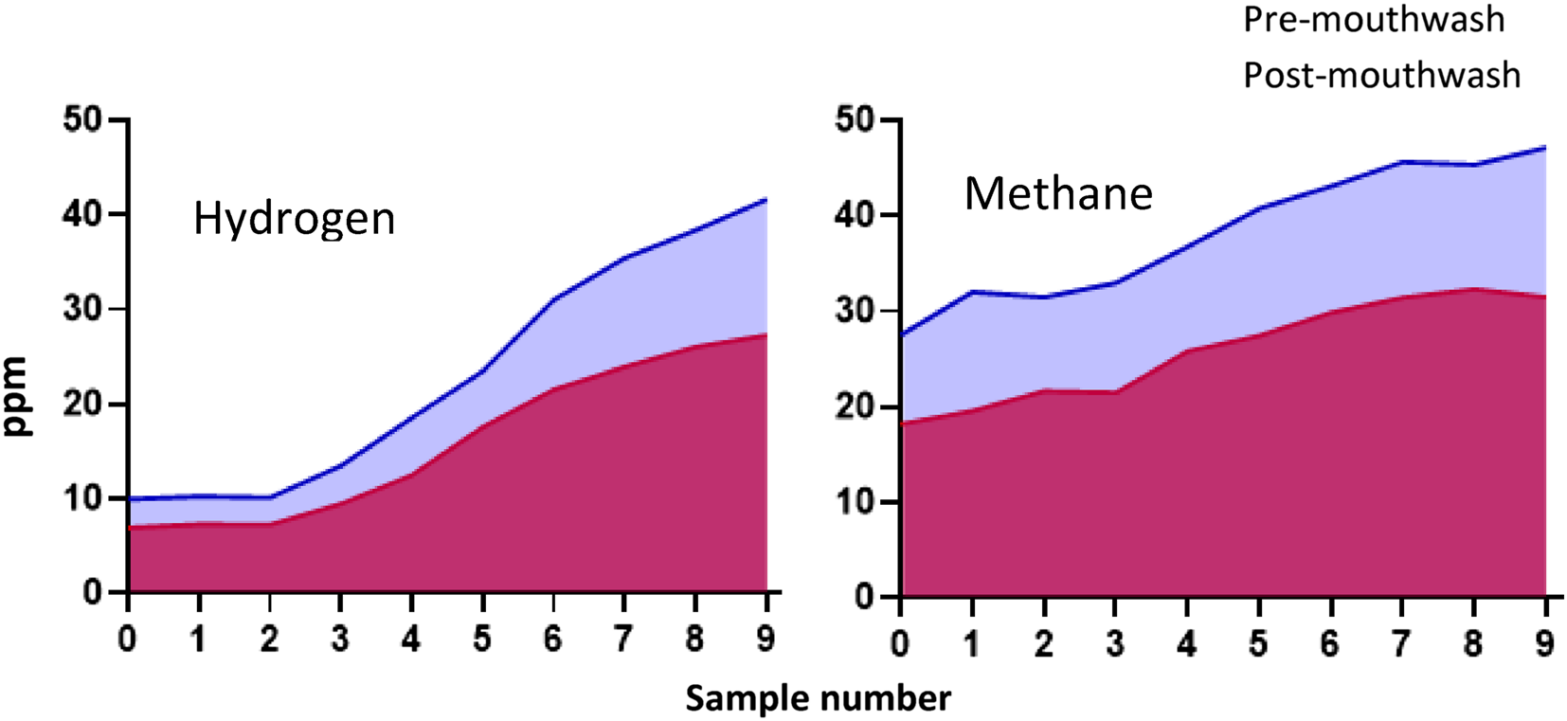
Mean AUC for hydrogen and methane pre versus post mouthwash (pooled data from 69 tests).

Figure 1 shows the AUC for pooled means of hydrogen and methane gas for each sample, demonstrating differences in pre- versus post-mouthwash gas produced.

When reviewing pre-mouthwash data, 97% of participants met “methane positive” criteria (≥ 10 ppm methane)^6,15^ at least once during the breath test. This was reduced to 91.3% by the mouthwash.

**Figure 2:**
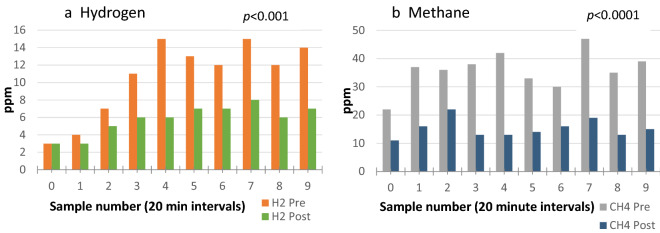
Changes in breath hydrogen (**a**) and methane (**b**) in one subject following chlorhexidine mouthwash at each time-point for the duration of breath testing. H_2_, hydrogen; CH_4_, methane; Pre, before mouthwash; Post, after mouthwash.

Decreases in hydrogen and/or methane following administration of the mouthwash were typically noted to occur throughout the duration of testing, as shown in the example graph below (Fig. 2) which presents data from a single subject.

**Figure 3:**
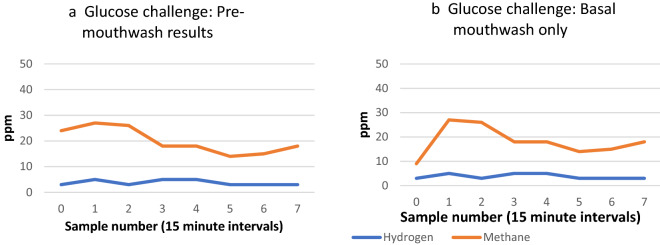
Negative breath test (**a**) becomes positive (**b**) following basal mouthwash during oral glucose challenge.

In the example shown in Fig. 3, a slight increase in methane occurred when “pre-mouthwash” data was observed (Fig. 3b). However, had a mouthwash been administered prior to commencing the test, a lower basal gas level (specifically methane in this example) would have been recorded and at the second sample (15 min later) a significant increase in methane (+ 18 ppm) occurs, suggestive of methanogenic overgrowth in the small intestine.

After calculating positivity for SIBO using the range of diagnostic criteria across all results, the mouthwash treatment did not alter the number of subjects satisfying the diagnostic criteria of an increase of 20 ppm of hydrogen, but significant differences were noted in meeting other criteria according to the oral preparation used, as presented in Table 6. Notably, “no mouthwash” versus “always mouthwash” did not show any significant differences across any of the diagnostic criteria, whereas meeting at least four of the criteria was more likely following single basal mouthwash compared to either “no mouthwash” or “always mouthwash”.

**Table 6:** Subjects meeting diagnostic criteria within 80 min depending on oral hygiene in 69 tests.

Criteria	Meets criteria for diagnosis: n (%)			Difference between 3 methods^a^	Difference between any 2 methods^b^		
	A. No (pre) mouthwash	B. Single T_0_ mouthwash	C. Always (post) mouthwash	A versus B versus C	A versus B	A versus C	B versus C
+ 20 ppm hydrogen over baseline	12 (17.4)	13 (18.8)	8 (11.6)	0.072	1.00	0.289	0.125
+ 12 ppm methane over baseline	24 (34.8)	42 (60.9)	17 (24.6)	< 0.001	< 0.001	0.118	< 0.001
+ 15 ppm combined over baseline	35 (50.7)	52 (75.4)	27 (39.1)	< 0.001	< 0.001	0.057	< 0.001
+ 10 ppm hydrogen btw 2 samples*	17 (24.6)	5 (7.2)	8 (11.6)	0.003	0.004	0.022	0.549
+ 10 ppm methane btw 2 samples*	26 (37.7)	38 (55.1)	24 (34.8)	0.004	0.012	0.804	0.009
+ 10 ppm hydrogen or methane btw 2 samples*	37 (53.6)	43 (62.3)	30 (43.5)	0.052	0.362	0.210	0.035

^a^Cochran’s Q test.

^b^McNemar’s test (alpha-value 0.016).

T_0_ = baseline.

*Between any 2 consecutive samples up to 80 min.

**Figure 4:**
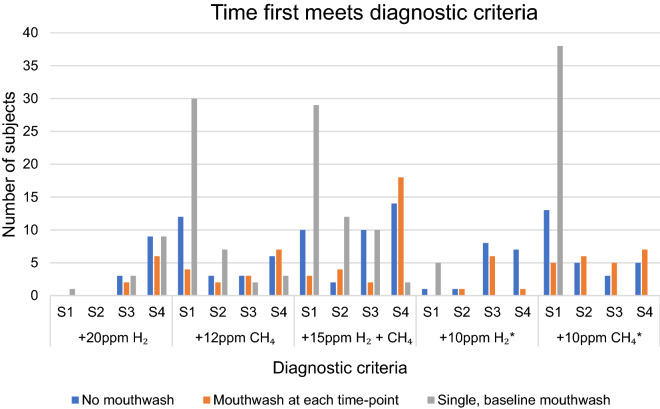
How a chlorhexidine mouthwash influences the time a subject meets various diagnostic criteria for small intestinal bacterial overgrowth during Hydrogen–methane breath testing. S, sample number post-baseline; H_2_, hydrogen; CH_4_, methane; *, between 2 sequential samples.

When evaluating how the various approaches to oral hygiene affect the time at which a breath test meets any of the criteria revealed substantial differences. These are represented in Fig. 4. With regards to the criteria of + 10 ppm hydrogen or methane between any two consecutive samples, data pertaining to “basal mouthwash only” is only possible for the first sample.

By evaluating results provided by the pre-mouthwash data at sample #1 (i.e., mimicking no mouthwash being given), 14 tests (20.3%) met at least one of the diagnostic criteria for SIBO. When examining data where baseline values are obtained after a mouthwash and compared to the first post-substrate sample (i.e., sample #1) collected before a mouthwash, 43 results (62.7%) met at least one diagnostic criteria, and one of the 14 became negative. Therefore, of these 43 tests, 30 (69.7%) would have been falsely interpreted as positive, had breath testing been conducted with a single mouthwash at baseline.

A positive breath test in 28 subjects (40.5%), based on pre-mouthwash data, would have been negated had a mouthwash been given prior to breath collection at baseline and sample #1. Using a “no mouthwash” scenario, 13 tests (18.8%) with negative results at sample #1 would be reclassified as positive if a single mouthwash were administered at baseline. Additionally, 39 tests (56.5%) which were negative from administering a mouthwash prior to each breath sample collection would have become positive if a mouthwash had been given at baseline only. Post hoc McNemar’s tests with Bonferroni correction (α = 0.016) showed significant differences between each of the pairs of methods (*p* < 0.001) as described in Table 2.

Applying the same construct to sample #4 after baseline (i.e., 80 min for all lactulose samples), if no mouthwash had been administered, some 13 positive tests (18.8%), would have become negative had a mouthwash been given prior to each breath sample collection. A single basal mouthwash would result in a change to positive in 18 tests (26%) that would have been negative had no mouthwash been administered at all. Similarly, 26, or 37.7% of tests with a negative result following mouthwash administration prior to collection of each sample would have become positive if a single mouthwash had been given at baseline. Post hoc McNemar’s tests with Bonferroni correction (α = 0.016) showed significant differences between diagnostic status change based on each method (*p* < 0.001), except the differences in altered status when comparing data based on pre-mouthwash versus basal mouthwash only and post-mouthwash versus basal mouthwash only were not statistically significant (*p* = 0.069).

While symptoms experienced by participants over the course of breath testing were recorded, these are not reported in this study.

## Discussion

This study demonstrates that the timing of oral preparation (mouthwash) for breath testing appears to impact the results upon which a diagnosis is made in a sizable proportion of subjects in whom basal breath hydrogen or methane is 10 ppm or more at baseline. Furthermore, the administration of a single mouthwash at baseline increases the likelihood of a positive diagnosis when compared to either no mouthwash at all or a mouthwash prior to collection of every breath sample throughout the duration of the breath test.

The findings of a significant contribution to methane concentrations from oral repositories was unexpected. Also surprising was the higher degree of modifiability of methane compared to hydrogen and the recovery of gas production within 20 min of each mouthwash.

In contrast to the well-absorbed glucose, the widely used lactulose is a synthetic disaccharide that is normally not metabolized nor absorbed in the small bowel. In a normal gut environment, a peak in breath gases is expected, typically at, or after 90 min^8^, due to fermentation of the substrate by colonic bacteria, with release of primarily hydrogen gas^17^. This may be excreted in the breath or metabolised by archae (phylum *Euryarchaeota*) to methane^18^. However, when there is microbial overgrowth in the small bowel, the test substrate is prematurely exposed to bacteria (or archae) creating an abnormal increase in gas production^1^. A peak that occurs before 90 min (assuming normal bowel transit time) has been suggested predictive of bacterial overgrowth in the small intestine^8,15^. As this study demonstrated, the timing of oral cleansing prior to commencing breath testing is crucial, with a single mouthwash at baseline being significantly associated with a positive diagnosis at the first sample post substrate ingestion.

The determination of what constitutes an appropriate diagnostic increase in a gas or gases and timing of these increases have varied over time and between reports and researchers. Despite the 2017 North American Consensus^8^ and the subsequent development of the American College of Gastroenterology (ACG) Clinical Guideline^15^, no universally accepted criteria currently exists. Both reports^8,15^ specified increases in hydrogen ≥ 20 ppm above baseline, and others suggest an increase of ≥ 12 ppm^4^, ≥ 10 ppm^3^ above baseline, or increases of at least 10 ppm over 2 consecutive samples^19,20^ as diagnostic of either SIBO or intolerance/malabsorption of lactose or fructose (according to the challenge sugar administered). Methane concentration ≥ 10 ppm at any time during testing has been declared as “methane positive”^8,15,21^, without addressing the potential for increases that occur in the small intestine, which were previously thought to be uncommon^22^. Definitions of diagnostic increases in methane also vary^23^; methane ≥ 12 ppm was the criteria in Mollar’s 2019 study^24^. While increases in methane were not detailed in the North American Consensus^8^, the ACG Clinical Guideline^15^ suggested a > 10 ppm increase in methane “requires confirmation” as an indicator of an overgrowth of methanogens. The 2009 Rome Consensus^5^ did not consider methane measurement would improve breath test accuracy, yet methanogens are able to convert hydrogen to methane^25^, resulting in lower excretion of hydrogen on the breath^18^. Thus, routine evaluation of methane and the specification of hydrogen-plus-methane ≥ 15 ppm within 90 min of testing to indicate SIBO is a reasonable consideration. This is reported in some testing centres^26,27^ and is the basis for inclusion as one of the diagnostic criteria in our analysis.

Prior to the ACG guideline, criteria for positivity have included increases of hydrogen or methane > 20ppm^28,29^, hydrogen ≥ 12 ppm above baseline^6^, or an increase of 12 ppm of hydrogen or methane where a basal concentration of ≥ 10 ppm was recorded^28^. Others have specified different increases depending on whether glucose or lactulose is the test substrate^30^. In some cases, it is stipulated that any increase should be sustained^30,19^.

While a range of criteria exists for time of determination, including 90–120 minutes^15^, the recommendation of a cut-off of 60–80 min by Sunny et al.^16^ and 60 min by Eisenmann et al.^3^ may improve specificity of the lactulose challenge. Current guidelines^8,15^ state a peak that occurs before 90 min (assuming normal bowel transit time) is suggestive of a bacterial overgrowth in the small intestine. The requirement of a further increase of at least 15 ppm hydrogen gas within 180 min as confirmation of colonic fermentation of lactulose has also been specified^16^ but this is not widely reported. Rapid oro-cecal transit has been demonstrated after test substrate ingestion^31,32^, yet slower small bowel transit has been demonstrated in the presence of methane^33^—a gas of particular interest in our findings. Generally, an early rise in breath gases following a lactulose challenge is accepted as a reasonable indicator of SIBO^20^. Considering this last point, our results demonstrate that the timing of oral cleansing prior to commencing breath testing may have an important impact on test results.

As pointed out by Siddiqui et al.^4^, the lack of comparability of breath tests for bacterial overgrowth poses challenges. The ACG Clinical Guideline^15^ (fundamentally based on the 2017 North American Consensus^8^, and compiled by many of the same group) sought to provide some consistency in terms of indications for breath testing, preparation of the patient, dose of carbohydrate, duration and intervals of testing, definitions of positive cut-offs, and treatment guidelines. However, other specifics such as total fluid volume consumed in the immediate period following substrate ingestion and preparation of the oral cavity are absent from these recommendations. The 2009 Rome Consensus^5^ also recommended a 15-s breath hold prior to expiration, which does not appear in either the 2017 or 2020 guidelines.

The lack of congruency regarding what constitutes normal fasting gas measurements for breath testing is discussed in our recent report^14^. One surveyed laboratory takes the following position regarding high fasting gas values: “This finding can be a result of consumption of poorly absorbable complex carbohydrates like potato, in the evening before the test. It can also be caused by small bowel bacterial overgrowth, or untreated celiac disease where there is enhanced exudation and prolonged fermentation of endogenous glycoprotein/exogenous carbohydrates. On the contrary, high fasting methane in methane producers normally is derived from endogenous rather than dietary substrates”^34^. In contrast a representative of another hospital-based laboratory stated “We do see a moderately elevated baseline that falls during the test frequently with methane. At first, I used to put a caveat in my reports to doctors that this could be due to inadequate preparation… however when we started to see it all the time, I began to accept this was the norm”^35^*.*

While fasting breath hydrogen ≥ 20 ppm has been reported in up to one third of patients presenting for breath testing^36^, we observed such concentrations in just 8 (2%) of the original cohort (n = 388); constituting 11.6% of the 69 meeting our inclusion criteria of ≥ 10 ppm of either gas at baseline. Rezaie et al.^37^ retrospectively reviewed over 14,000 breath tests and suggested fasting methane ≥ 5 ppm to be predictive of “excessive” methane production, i.e., methane ≥ 10 ppm at any time during breath testing. Applying a ≥ 10 ppm cut-off resulted in methane-producers comprising 14.4% of our whole-cohort, similar to the 15% reported by Romagnuolo in 2002^36^ and Rezaie in 2015^37^. The administration of a mouthwash at baseline in our study resulted in a reduction in expired methane to below the 10 ppm threshold in 9 (13%) of the 69 included subjects or 8.4% of those with basal methane ≥ 10 ppm.

Early reports suggested that methane is excreted on the breath of about one third of adults^38^, around 20% of those with IBS^29^ and low production (less than 4ppm^17^, or up to 10 ppm) the expected norm^39^.

The four methanoarchaeal species most commonly reported in humans, including in feces, vaginal mucosa and subgingival plaque are *Methanobrevibacter smithii, M. oralis, Methanosphaera stadtmanae,* and, more recently, *Methanomassiliicoccus luminyensis*^40^ which is more common in the elderly^41^. *Methanobrevibacter smithii* is the predominant methanogen inhabiting the human colon in 15–30%^6^, and possibly up to 95.5% of individuals^22,42,43^ and is expected to be the primary source of methane gas in humans^22^. *Methanosphaera stadtmanae* has been detected in the stool of 29.4% of people^44^, and antigenically similar organisms have been isolated from dental plaque^45^. *Methanobrevibacter oralis* and other organisms phylogenetically similar to *M. ruminantium* and *M. aboriphilus* have been isolated from root canal infections^46^, and *M. oralis* from infected periodontal implants^47^. Lepp et al.^48^ reported *M. oralis* was only present in those with severe periodontal disease, but Grine et al.^49^ described *M. oralis* and *M. smithii* in the oral fluid of tobacco smokers with no clinically detectable disease of the oral cavity. DNA from other methanogens has been detected in some individuals^44^, and in addition to *M.oralis, M. smithii, Methanobacterium congolense, Methanosarcina mazei* and *Candidatus methanomethylophilus sp.* have been isolated from the human oral cavity^41^. The liberation of methane by *Clostridia butyricum, some strains of C. perfringens, C. septicurn, C. histolyticum* and *C.difficile, as well as by Bacterioides* species including *B. thetaiotaomicron* has also been described^50^. Belda-Ferre et al.’s study^51^ revealed methanogens belonging to the classes *Methanopyri, Methanomicrobia* and *Methanococci* in addition to *Methanobacteria* in the oral microbiota^51^.

Methanogens are strictly anerobic^22^, which is consistent with ability to survive in sub-gingival tissues, deep infections and root canals. It would not be expected that any gases produced from these sites would substantially reach the mouth, or at least be rapidly modified by a mouthwash, as seen in our study. This rapid recovery of gas production suggests a more superficial repository of methanogens, possibly the tongue, as has been suggested by the results of work by Amou et al.^52^ and Alqumber et al.^53^, or in saliva, which has been reported in smokers^49^. Both have implications for translocation of archaea and bacteria into the gastrointestinal tract.

Data suggest a relationship between poor oral health and gastric ulcers, gastrointestinal cancers and hepatic diseases^51,54,55^. Methanogens have been identified in subjects with oral pathologies^56^ and it is probable that the oral microbiota influences the gut microbiome^54^. We are unable to identify any research investigating a relationship between the oral microbiota and functional gastrointestinal disorders or SIBO.

The finding of the rebound effect following oral cleaning at baseline raises a procedural concern. We demonstrated that this “rebound” may result in recovery of gas production by the first subsequent breath sample, such that a diagnosis of overgrowth, or saccharide intolerance/malabsorption may be made. However, if a second mouthwash were to be given, this rise in gas is, in most cases, annulled.

In the light of these findings, the administration of both a single mouthwash and cleaning of teeth prior to commencing breath testing, as recommended by Ghoshal^6^, and conducted in the 2018 study by Wilder-Smith et al.^57^ may have contributed to a falsely low baseline in at least a portion of their subjects, with rebound recovery of gas production affecting interpretation of subsequent data. Data pertaining to basal gases were not presented in their report.

We have demonstrated that, in addition to hydrogen production, methanogenesis may originate in the oral cavity of many individuals presenting for hydrogen–methane breath testing. A question arises as to the role of the implied overgrowth, or dysbiosis, of oral bacteria, including methanogens, in the gastrointestinal symptoms for which patients are referred for testing. While it is logical to reduce the risk of falsely elevated baseline gases by administering an antiseptic mouthwash prior to breath testing, this study demonstrates that false positive results, and possibly erroneous diagnosis of overgrowth of bacteria or methanogens in the small intestine may be a consequence.

In practice, a range of diagnostic criteria are applied to the interpretation of breath tests. While the use of increases of 12 ppm for methane and 15 ppm for combined gases are not widely published they are utilized in research^23^, as well as in practice^26^. We chose to evaluate results based on what is both in the published literature and used in laboratory interpretations. While the standard for hydrogen–methane breath testing is evaluation of breath samples exhaled via the oral cavity, variation in detected levels of volatile gases from oral versus nasal expiration have also been reported^58^.

A strength of this study is the strict low-residue diet, ensuring all participants presented with minimal ongoing colonic fermentation. The selection of a well-established oral antiseptic mouthwash and the timing of delivery to replicate what is in current protocol in some clinics^14^ allows the comparison of this procedure to other scenarios.

The study design has demonstrated the rapid recovery of oral microbiota, with gas production, within 15–20 min of a chlorhexidine mouthwash. As far as the authors can ascertain, this has not previously been reported.

Characteristics of our cohort is limited to gender, weight and age. Other factors may increase the risk of developing SIBO, such as obesity^59^, which is also associated with periodontal disease^60^. While measures of body weight were collected (Table 1), absent height data prevents calculation of body mass.

No clear definition exists for determination of elevated baseline breath gases. Therefore, our set-value of 10 ppm of either hydrogen or methane gas as being “elevated” may be considered arbitrary. For example, a combined value of 10 ppm may also constitute an elevation, but whether this is clinically relevant, or if the cut-off should be lower or higher, is currently unknown. Likewise, designating a minimum reduction of breath gases of 25% or 10 ppm as inclusion criteria may also be considered arbitrary given the lack of evidence to support this.

We did not include a control arm; and the determination of change in breath gases due to oral lavage with water as a control may be useful. If saliva is a repository of flora that produce gases, simply drinking water and/or swallowing prior to collection of individual breath samples may also influence test results. If this were to be the case a major change in breath test protocol would be required.

We collected a single baseline breath sample from each participant, which may be considered a limitation. Some researchers suggest that the average of two^5^ or three-four^6^ baseline breath samples should be collected, and the average of these values used to determine fasting breath hydrogen. As far as the authors can determine, no investigation has been conducted to determine best practice in this regard.

This study suggests that the oral microbiota contributes to measurements of breath gases. In up to 30% of patients with at least one basal gas ≥ 10 ppm this may alter test outcomes and diagnosis, and therefore treatment. The relationship between oral bacterial overgrowth and functional gastrointestinal disorders beyond GI cancers and ulcers requires a thorough examination. Further rigorous investigation is recommended to determine oral contributors to breath gases in subjects presenting with gastrointestinal symptoms; best practice for preparation of the oral cavity; and/or establishing a procedure that identifies those subjects in whom basal gas elevations are modifiable. Until further data are available, we recommend the collection of two baseline samples when conducting breath testing—one before, and one after a mouthwash. This practice will determine the likely contribution of oral microbiota in subsequent breath gas measurements and has the potential to add valuable information to the clinical dataset for patients. Those facilities unable to incorporate a protocol with pre- and post- mouthwash samples at baseline should refrain from advising oral cleansing within at least 30 min of commencement of breath testing.

## Conclusion

Baseline elevations of expired hydrogen or methane seen in breath testing may be due to the oral microbiota, including methanogens. Variations in gas production such as those seen in this study has significant implications on test interpretation and subsequently on diagnosis.

The relationship between oral dysbiosis and gastrointestinal symptoms is under-explored. The prospect that identification and treatment of the oral microbiota may alter patient outcomes is an interesting possibility that warrants investigation.

Examination of the influence of various oral hygiene practices on breath test results in a larger cohort is recommended to determine best practice for addressing this issue in clinical settings.

## Data Availability

Data will be made available upon reasonable request.
